# Volatile Organic Compounds in Anatomical Pathology Wards: Comparative and Qualitative Assessment of Indoor Airborne Pollution

**DOI:** 10.3390/ijerph14060609

**Published:** 2017-06-07

**Authors:** Massimo Cipolla, Alberto Izzotti, Filippo Ansaldi, Paolo Durando, Maria Teresa Piccardo

**Affiliations:** 1 Mutagenesis Unit, IRCCS AOU San Martino IST, Genoa 16132, Italy; massimo.cipolla@hsanmartino.it (M.C.); izzotti@unige.it (A.I.); 2 Department of Health Sciences, University of Genoa, Genoa 16132, Italy; ansaldi@unige.it (F.A.); paolo.durando@unige.it (P.D.); 3 Hospital Direction, IRCCS AOU San Martino IST, Genoa 16132, Italy; 4 Occupational Medicine Unit, IRCCS AOU San Martino IST, Genoa 16132, Italy

**Keywords:** VOCs, indoor pollution, human health

## Abstract

The impact of volatile organic compounds (VOCs) on indoor air quality and on human health is widely recognized. However, VOC contamination in hospital indoor air is rarely studied and chemical compounds that singularly do not show high toxicity are not submitted to any regulation. This study aimed to compare VOC contamination in two different anatomical pathology wards in the same hospital. Hydrocarbons, alcohols, and terpenes were sampled by passive diffusive samplers. Analytical tests were performed by thermal desorption coupled with gas chromatography and mass spectrometry detector. Results highlighted a different VOC pollution in the two wards, due to the structural difference of the buildings and different organizational systems. The scarcity of similar data in the literature shows that the presence of VOCs in pathology wards is an underestimated problem. We believe that, because of the adverse effects that VOCs may have on the human health, this topic is worth exploring further.

## 1. Introduction

Healthcare workers may be exposed to a wide range of chemicals emitted from laboratory and pharmaceutical activities and from different substances such as disinfectants, sterilizing agents, and anesthetic gases [1]. In a hospital environment, histopathology laboratories represent a separate reality for type and quantity of reagents utilized, spanning from organic solvents to histological preserving agents. Mixtures of volatile organic compounds (VOCs) are utilized in these laboratories in large quantities, even about 10 liters per week, and also in environments characterized by limited air volumes.

Several studies have evaluated the concentrations of formaldehyde, recognized as a human carcinogen [2] and accordingly regulated [3], in histopathology laboratories and the association between formaldehyde and genotoxic effects in workers [4,5,6,7]. Conversely, very few studies have been conducted on VOC contamination in hospitals [1,8,9] and chemical compounds that singularly do not exhibit high toxicity—such as heavy alcohols, alkanes, and terpenes—are not subject to control. However, some of them have shown evidence of adverse effects on human health: occupational exposition to ethanol vapor can increase urinary ethanol biomarkers [10]; acute inhalation exposure to isopropanol can cause central nervous system depression due both to isopropanol itself and to its conversion to acetone, its primary metabolite [11]; xylene, commonly used to deparaffinize tissues before staining or extracting DNA, has been considered occupationally hazardous because its inhalation can affect various organs such as eyes, nose, throat, lungs and it can compromise the respiratory function and the central nervous system [12]; also teratogenic effects of xylene exposure have been described in animals [13]; d-limonene, when it undergoes a reaction with ozone, produces a number of oxidation products that can cause airway effects on humans [14]. Moreover, low concentrations of complex mixtures of VOCs may increase the risk of sick building symptoms (SBS) in workers [15], causing irritation of the eyes, skin, respiratory tract, central nervous system, and viscera [16]. The synergic interaction between VOCs and different chemical structure and physico-chemical properties can alter pharmacokinetics processes, including their absorption, distribution, metabolism, and excretion, inducing toxicity [17]. For all these reasons, monitoring levels and composition of the new pollutants mixture would be beneficial in order to evaluate their effects on human health.

The aim of the study was a comparative and qualitative investigation of VOCs in two different anatomical pathology wards of the same hospital in view of the reorganization of the laboratories and their unification into a single unit.

## 2. Materials and Methods

### 2.1. Study Area

The study area is located in two different buildings of S. Martino Hospital (IRCCS-AOU San Martino—IST) in Genoa, Italy, a Comprehensive Cancer Center recognized by the Organization of European Cancer Institutes, including both the Hospital and the University. The complex structure of the Institute, with an area of 340,000 m^2^ and 12 km of roads inside, is a little town near the city center and counts 5000 healthcare workers. The first anatomical pathology ward (A) is located on the second and third floor of a building from the first half of the 20th century, characterized by high ceilings and large rooms. Laboratories are on the second floor. The second ward (B) resides on the second floor of a building built in the 1980s, and it is characterized by lower ceilings and smaller rooms. In the two wards, the activities were comparable and the working volume was proportional to the size of the structure and the number of staff employed. In both wards, the following sampling areas were selected: the “processing room”, where above all automated processes occur, dehydration, diaphanization and embedding; the “histology room” for inclusion, microtomy, and staining slides; and the “slides storage room” for slides archive. The “secretariat room”, adjacent to the respective laboratories, has been chosen as control.

### 2.2. VOC Sampling

PerkinElmer stainless steel sampling tubes (Perkin Elmer Italia S.p.A., Milan, Italy) pre-packed with Chromosorb 106 (60/80 mesh), previously conditioned [18] were positioned in sampling areas for passive VOC monitoring, out to the direct sources, in the middle of the room or on the opposite wall. Samplings were performed for seven days, from 6 to 13 August 2015, in ward A and from 12 to 17 August 2015 in ward B.

For each sampling, two unexposed samplers have followed the same procedures of the exposed tubes to be used as the basis of reference to zero exposure (four blanks).

VOC sampling has followed UNI EN ISO 16000-5 and UNI EN ISO 16017-2 regulations.

### 2.3. VOC Analyses

VOC samples were analyzed by thermal desorption (TurboMatrix 650, Perkin Elmer Italia S.p.A.) coupled with a gas chromatograph (Clarus 500, Perkin Elmer Italia S.p.A.) and a capillary column Rxi-1 MS 60 m, 0.25 mm id, 1.0 um film thickness (Superchrom Cod. No. 13356, Restek, Rome, Italy) confluent in a mass detector (Clarus 560 S, Perkin Elmer Italia S.p.A.).

Thermal desorption of the sorbent tubes was carried out at 220 °C with a flow rate of 40 mL min^−1^ of He for 15 min (primary desorption), during which time the eluted VOCs were transferred from the tube to a cryogenic internal trap (Tenax^TM^ TA, Perkin Elmer Italia S.p.A.) maintained at −20 °C. After primary desorption, the cold trap was rapidly heated from −20 °C to 300 °C (secondary desorption) and then maintained at this temperature for 6 min. During the secondary desorption, VOCs were injected onto the capillary column, applying a flow of 2 mL min^−1^. The column oven temperature was initially 32 °C for 7 min, increased to 120 °C at a rate of 3 °C min^−1^, then increased to 250 °C at a rate of 9 °C min^−1^, and finally maintained at 250 °C for 5 min.

The acquisition of the analysis for scanning the total ions was made necessary in view of the composition of unknown samples; the comparison between samples was carried out using, for each compound, the main ion in the spectrum of every single chromatographic peak or the better discriminant ion from the possible confounders.

For the quantification of benzene, toluene, ethylbenzene, and *m*-,*p*-,*o*-xylene (BTEX) a calibration curve was built, injecting at standard conditions for temperature and pressure (EPA/NIST, 20 °C—1 atm) by injector ATIS-adsorbent tube injector system (Supelco–Sigma Aldrich S.p.A., Milan, Italy), flux: 85 mL/min × 4 min. each tube) in the tube samplers containing 1, 2, 5, and 10 mL of a gas mixture of environmental pollutants (Restek P/N 34420-PI, mix, 1 ppm in nitrogen, 104 L;1800 psi) at a concentration of 1 ppm in ultra-pure nitrogen, using gastight syringes (0087705MR-VLLMA-GT, SGE Analytical Science-Chebios, Rome, Italy).

Quantification of BTEX was performed using diffusion coefficients experimentally obtained [18]. The results have been corrected for temperature, pressure, and sampling duration. Method detection limit (MDL) for each BTEX was estimated on the basis of the mean of background signal plus three times the standard deviation (mean 4 blank signals + 3 standard deviation (SD)) MDL values for BTEX ranged from 0.019 to 0.04 µg/m^3^. The limit of quantification (LOQ) defined as 3 × MDL was 0.1 µg/m^3^ for each BTEX. Blanks analyzed were always at background signal levels.

### 2.4. Statistical Methods

To analyze the relationship between VOCs, a linear regression model was used [19]. R^2^ was utilized as a measure of fit.

## 3. Results

Different classes of compounds have been qualitatively examined: alcohols, esters, ketones, terpenes, and hydrocarbons (aromatics, aliphatic, heterocyclic, and polycyclic).

**Figure 1:**
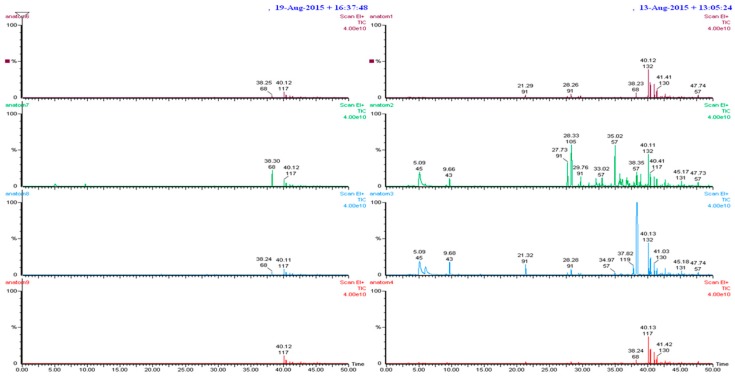
Chromatograms of VOCs measured in the “histology rooms” of anatomical pathology wards A (**right**) and B (**left**). The numbers above each peak indicate, from top to bottom, elution time and ion (*m/z*). Some peaks are deformed by the high amounts present.

Table 1 reports VOCs detected and listed according to elution time. Two examples of VOCs chromatograms are shown in Figure 1.

**Figure 2:**
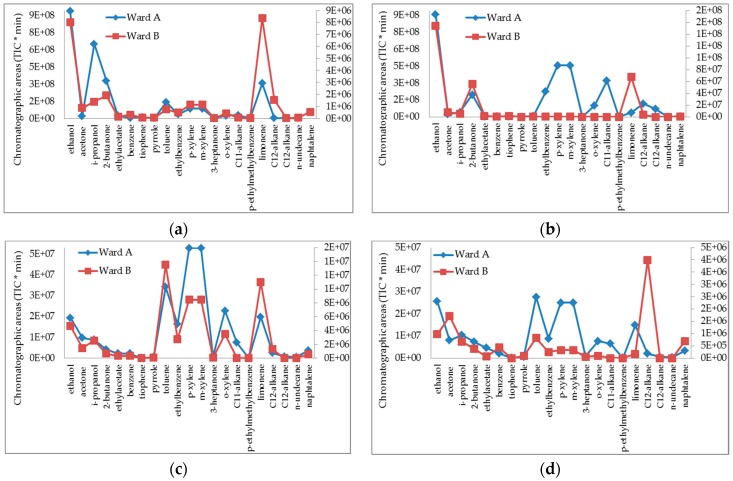
Chromatographic areas (TIC/min, total ion current per minute) of detected compounds: comparison between different sampling sites of the two anatomical pathology wards (A, B): (**a**) histology room; (**b**) processing room; (**c**) slide storage room; (**d**) secretariat room. The scale to the left refers to the blue line and the right one to the red line.

Figure 2 shows the chromatographic areas of the main molecules detected in the different places of the two wards, in order of gas-chromatographic elution time, volatility, classes of compounds, and molecular weight.

All examined compounds were higher in ward A, especially in the “histology room” and “processing room” laboratories. In particular, A areas subtended >100 times as compared to ward B for ethanol, isopropanol, 2-butanone, ethylacetate, thiophene, toluene, *p*-ethylmethylbenzene. In the “histology room”, limonene and the aromatic compounds—xylene and ethylbenzene—were also higher in ward A than in ward B (70 and 36 times greater respectively). Similarly, in the “processing rooms”, chromatographic areas of xylene and ethylbenzene were much higher in ward A than in ward B, as well as alkanes used as clearing agents. The secretariats, adjacent to the laboratories, showed similar discrepancies between ward A and B.

The linearity of the relationships between ethanol and butanone or thiophene were calculated by linear regression analysis obtaining the following results: (a) ethanol and 2-butanone R^2^ = 0.9277 (*p* < 0.01); (b) ethanol and thiophene R^2^ = 0.6751 (*p* = 0.012), as shown in Table S1 (Supplementary Materials).

**Figure 3:**
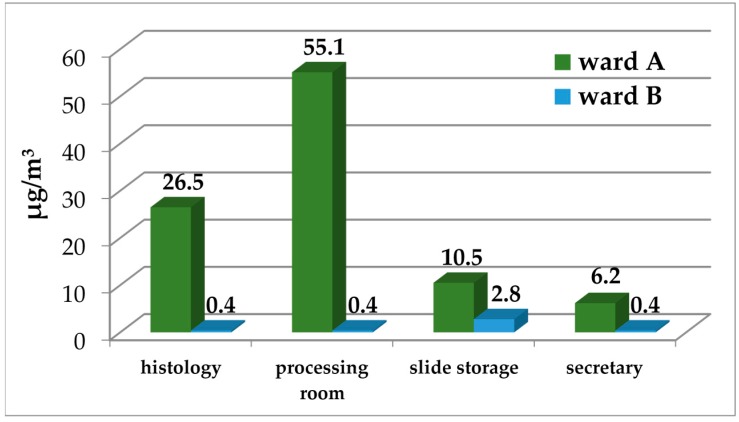
∑BTEX concentrations (µg/m^3^) in the different places of two anatomical pathology wards (A, B).

Figure 3 reports sum of benzene, toluene, ethylbenzene and xylenes (∑BTEX) concentrations measured in the different areas. Benzene levels were at the limit of quantification (LOQ) in all sites. The concentrations of toluene, ethylbenzene, and xylenes were higher in ward A than in ward B, where each compound was almost always at LOQ or below LOQ except in “slide storage”, where low concentrations of these compounds were detected.

Difference between ∑BTEX mean values of sampled areas in ward A (24.6 ± 22.1 µg/m^3^) and B (1.0 ± 1.2 µg/m^3^) were statistically significant (*p* = 0.023).

## 4. Discussion

The main molecules detected in this study were part of the standard composition of the solvents used for processing histological samples, from fixation to mounting slides; other molecules, less immediately ascribable to the methods used, are contaminants of the reagents used. Typically, they include the main compounds contained in a denaturant "red" ethyl alcohol: 2-butanone (methyl ethyl ketone) and thiophene. This interpretation may be supported by the linear trend between ethanol and 2-butanone or thiophene. In the past, xylene has been used in the histology laboratories during tissue processing, to deparaffinize sections before staining and mounting slides during covers lipping. Xylene hazardous effects by inhalation, including depression of the central nervous system, irritation of nose throat and lungs [12,20], led in large part to its substitution. Currently, the clearing agents used in histology include isopropanol, used alone or mixed with molten paraffin, alkanes and terpenes [21], all compounds detected in this study.

The volatility of these compounds justifies their presence, for dispersion effect, in all environments of ward A, including the control room.

About BTEX, only xylene was a reagent used in small amount in the laboratories of anatomical pathology. Ethylbenzene and toluene, not used as reagents but present in the different rooms of ward A, might be derived from other chemicals. BTEX pollutants also originated from urban vehicular traffic. As shown in several studies, when BTEX are coming from traffic they are well related each other [22,23]. In our study, outdoor BTEX sources cannot be excluded too, especially in ward A where the windows were always open. Anyway, BTEX concentrations measured by us were not different from those found in European indoor environment [24], as well as from those measured by Bessonneau et al. [1] in different hospital sites.

The comparative analyses of the two wards highlight a different VOC pollution due to the structural difference of the buildings and the different organizational system. In particular, it can be assumed that well-defined elements contributed to the best air quality in ward B including: (a) an automatic system to deparaffinize sections before slides staining and mounting; (b) the fume hoods still existing but less used because of being replaced by automated systems; (c) proper thermal conditioning with adequate air changes (10/h) and temperatures suitable for an analysis laboratory (measured temperature 23.5 ± 1 °C). On the contrary, in ward A, the following conditions contributed to the worse air quality as compared to ward B: (a) manual staining with baskets holder slides and trays mounting; (b) the fume hoods used for slide manipulation and inclusion had little depth and were often kept opened because of the large size of the equipment contained (paraffin inclusion unit); (c) inadequate thermal conditioning causing temperatures unsuitable for the laboratories (measured temperature 27 ± 4 °C); these high temperatures further favored VOC volatilization contributing to their dispersion in the air. The inadequacy of the workplaces can contribute to increasing the chemical hazard within the anatomical pathology laboratories as found by a previous study where pathology laboratory technicians inappropriately exposed to organic solvents had developed increased levels of DNA damage [25]. However, for organizational reasons, the two wards were provisionally gathered together in the largest structure, with ward A now including ward B. The aim of this process was also to place air purifiers in order to improve the safety of workers.

The strength of our study is recording additional insights concerning indoor microenvironments in anatomical pathology wards, where there is lack of data.

The main limitation of our study is the small size of our samples and the brief period of observation, that was, only one week in the summer. The levels of pollutants could differ in other periods—especially in ward A—due to seasonal effects, but the change of scenario has not made possible further comparisons.

## 5. Conclusions

At present, the importance of VOCs in air contamination and human health is recognized. Biosafety practices in anatomical pathology wards are crucial to preventing unnecessary exposure to both chemical and biological substances. However, our preliminary data show some critical issues that might not be an isolated case in hospital environments, especially considering the scarcity of studies similar to those herein presented. The lack of data in the literature shows that the presence of VOCs in pathology wards is an underestimated problem. We believe that, for the repercussions that VOCs may have on human health, this topic is worth exploring further.

## Figures and Tables

**Table 1:** Volatile organic compounds (VOCs) detected and listed according to gascromatographic elution time.

VOC	Retention Time (min)	VOC	Retention Time (min)
Ethanol	4.96	m-xylene *	28.27
Acetone	5.58	3-heptanone	28.79
*i*-propanol	5.93	*o*-xylene	29.76
2-butanone	9.69	C11-alkane	34.94
ethylacetate	11.01	*p*-ethylmethylbenzene	37.61
benzene	14.08	Limonene	38.61
thiophene	14.42	C12-alkane	38.35
pyrrole	19.74	C12-alkane	38.93
toluene	21.30	*n*-undecane	41.02
ethylbenzene	27.71	naphthalene	43.55
*p*-xylene *	28.27		

* Coelution.

## Supplementary Materials

The following is available online at www.mdpi.com/1660-4601/14/609/s1: Table S1: VOCs detected and listed according to gas-chromatographic elution time.
