# Supplementary material for: Volatile Organic Compounds in Anatomical Pathology Wards: Comparative and Qualitative Assessment of Indoor Airborne Pollution
Source: Int J Environ Res Public Health. 2017 Jun 7;14(6):609. doi: 10.3390/ijerph14060609 (PMC5486295; doi:10.3390/ijerph14060609)
Supplement: Supplementary file 1 [file ijerph-14-00609-s001.pdf]

Table S1. Chromatographic areas (measured as TIC/min, total ion current per minute) of ethanol, 2-butanone and thiophene measured in ward A and ward B; reciprocal relationships between ethanol and other compounds measured by regression analysis (R^2).

	**ethanol**	**2-butanone**	**thiophene**
Slide storage room "A"	19309628	4160136	2216
Processing room "A"	905910016	196863376	3007932
Hystology room "A"	933407488	326647264	12206169
Secretariat room "A"	25591334	7440968	172652
Slide storage room "B"	4643855	662437	30899
Processing room "B"	154108664	55432104	1597099
Hystology room "B"	8016267	1911267	52572
Secretariat room "B"	976529	381601	3427
	ethanol: 2-butanone	$R^2 = 0.9277$	$p < 0.01$
	ethanol: thiophene	$R^2 = 0.6751$	$p = 0.012$
